# Changes in Cardiac Function and Exercise Capacity Following Ferric Carboxymaltose Administration in HFrEF Patients with Iron Deficiency

**DOI:** 10.3390/diagnostics15151941

**Published:** 2025-08-02

**Authors:** Anastasios Tsarouchas, Constantinos Bakogiannis, Dimitrios Mouselimis, Christodoulos E. Papadopoulos, Efstratios K. Theofillogiannakos, Efstathios D. Pagourelias, Ioannis Kelemanis, Aristi. Boulmpou, Antonios P. Antoniadis, Nikolaos Fragakis, Georgios Efthimiadis, Theodoros D. Karamitsos, Vassilios P. Vassilikos

**Affiliations:** 1 Third Cardiology Department, Hippokrateion General Hospital, School of Medicine, Aristotle University of Thessaloniki, GR54642 Thessaloniki, Greece; 2 Second Cardiology Department, Hippokrateion General Hospital, School of Medicine, Aristotle University of Thessaloniki, GR54642 Thessaloniki, Greece; 3 First Cardiology Department, AHEPA University Hospital, School of Medicine, Aristotle University of Thessaloniki, GR54642 Thessaloniki, Greece

**Keywords:** HFrEF, ferric carboxymaltose, strain imaging, iron deficiency, exercise capacity, diastolic dysfunction

## Abstract

**Background/Objectives:** Iron deficiency (ID) is a common and prognostically relevant comorbidity in heart failure with reduced ejection fraction (HFrEF). It contributes to reduced functional status, exercise capacity, and survival. Intravenous ferric carboxymaltose (FCM) improves symptoms, but its effect on cardiac structure and function remains incompletely understood. The aim of this study was to assess the impact of intravenous FCM on echocardiographic indices of left ventricular (LV), left atrial (LA), and right ventricular (RV) morphology and function in HFrEF patients with ID and determine whether these changes correlate with improvements in exercise capacity. **Methods:** This sub-analysis of the RESAFE-HF registry (NCT04974021) included 86 HFrEF patients with ID (median age 71.8 years, 83% male). Transthoracic echocardiography was performed at baseline and 12 months post-FCM. Parameters assessed included LV ejection fraction (LVEF), LV global longitudinal strain (GLS), LV diastolic function grade, LAVi, LA strain, TAPSE, and RV free wall strain (FWS). Peak VO_2_ was measured to assess exercise capacity. **Results:** LVEF improved from 29.3 ± 7.8% to 32.5 ± 10.6% (*p* < 0.001), LV GLS from −7.89% to −8.62%, and the LV diastolic dysfunction grade improved (*p* < 0.001). LAVi, peak LA strain, TAPSE, and RV FWS also showed significant improvement. Peak VO_2_ increased from 11.3 ± 3.2 to 12.1 ± 4.1 mL/min/kg (*p* < 0.001). Improvements in LVEF, RV FWS, and LV GLS were independent predictors of VO_2_ increase (*p* < 0.001, *p* < 0.001, and *p* = 0.01, respectively), explaining 42% of the variance. **Conclusions:** FCM therapy improves biventricular and atrial function, with echocardiographic gains correlating with an enhanced exercise capacity in HFrEF patients with ID.

## 1. Introduction

Heart failure (HF) is a complex clinical syndrome characterized by the heart’s inability to meet the metabolic demands of the body, typically due to impaired cardiac output or elevated filling pressures. It is estimated that 23 million people globally suffer from heart failure, and heart failure with reduced ejection fraction (HFrEF) amounts to 50% of cases [1]. The gravity of this diagnosis is unquestionable; even in modern cohorts, the 1-year mortality rate of HFrEF patients on optimal treatment is 15–25% [2].

Transthoracic echocardiography (TTE) is an invaluable imaging modality in the workup and prognostication of HF. As the usual first means of quantifying patients’ left ventricular ejection fraction (LVEF) [3,4], TTE enables the quantification of several structural and functional anatomic parameters of the cardiac muscle with significant prognostic value [5]. Left ventricular systolic dysfunction is ubiquitous in patients with HFrEF and can be best quantified with parameters such as the LVEF and, more recently, the left ventricular (LV) global longitudinal strain (GLS). The latter is still comparatively laborious to derive from 2D-TTE views; however, it has proven superior to risk stratification in HFrEF [6]. Additionally, the LV outflow tract velocity time integral can serve as a non-invasive method to assess cardiac output and can provide significant insight in LV systolic function, especially in cases of advanced HF [7].

Diastolic dysfunction is invariably present in HFrEF, and the extent of impairment can be quantified using routinely measured parameters, such as the left atrial volume, the E/A ratio, and the E-wave deceleration time [8,9], as well as the ratio of the transmitral early LV filling velocity to the early diastolic TDI velocity of the mitral annulus (E/e′) [10]. Together, these variables can be used to detect and classify LV diastolic dysfunction in Grades I-III, where Grade I is the least-severe and Grade III corresponds to the most-severe dysfunction [11]. Strain imaging also enables the quantification of the LV diastolic strain rate, another experimental marker of myocardial relaxation, a crucial component of LV diastolic function [12].

Although HFrEF is synonymous with left ventricular dysfunction, it can lead to, and in turn can also be affected by, alterations in the form and function of the left atrium, as well as the right ventricle. The left atrial diameter (LAD), left atrial volume index (LAVi), the left atrial emptying fraction (LAEF) are traditional indices of the left atrial status [13], while the left atrial function index (LAFi) [14] and the peak left atrial strain (LAS) [15] are relatively novel modalities with potential value in the prognostication of HFrEF. Regarding the right ventricle (RV), apart from cases where RV functionality is directly hampered by the injury that caused LF dysfunction, such as an inferior myocardial infarction [16], the increased afterload brought about by pulmonary congestion secondary to LV dysfunction can lead to deterioration of RV functional indices [5]. RV fractional area change (RV FAC) and tricuspid annular plane systolic excursion (TAPSE) are sensitive markers of RV dysfunction and an increased mortality risk [17]. Additionally, RV free wall strain is a relatively new marker with great promise in the risk stratification of HFrEF [18].

Iron deficiency (ID) is increasingly recognized as a source of significant morbidity and mortality in HFrEF [19]. Indeed, several studies have established that ID is an independent predictor of mortality [20,21] and a risk factor for a poor exercise capacity and functional status in patients with HFrEF [22,23]. Furthermore, ID is disconcertingly frequent in HFrEF; more than half of patients with HFrEF fulfill the criteria for ID as defined by the European Society of Cardiology [24]: a serum ferritin value of less than 100 ng/mL, or serum ferritin less than 300 ng/mL with a concurrent transferrin saturation (TSAT) less than 20% [25,26]. According to the latest literature, in patients with HFrEF, absolute iron deficiency is defined as reduced ferritin levels (<100 ng/mL), while functional iron deficiency is defined as a TSAT < 20% [27].

Intravenous iron supplementation with ferric carboxymaltose (FCM) has repeatedly demonstrated promising safety and efficacy in the treatment of ID in HFrEF [28,29,30,31,32,33] and is presented by the 2016 and 2021 ESC guidelines as the preferred treatment option for ID in symptomatic patients with HFrEF [25]. The precise physiological mechanism behind the improvement in patient symptomatology and exercise capacity is not entirely elucidated; our team has previously posited that the restitution of intra-cardiomyocyte iron content can improve their metabolic profile and eventually lead to increased cardiomyocyte electrochemical stability [34,35].

Our team further hypothesized that this would lead to an improvement in myocardial function and a reduction in life-threatening ventricular arrhythmias in patients treated with ferric carboxymaltose. The effect of FCM on arrhythmogenicity was assessed with the Iron Intravenous Therapy in Reducing the burden of Severe Arrhythmias in HFrEF (RESAFE-HF), a prospective, single-center, open-label registry study (NCT04974021).

The present work presents a sub-study of RESAFE-HF that aimed to investigate the effect of IV FCM on conventional and novel ultrasonographic indices of left ventricular (LV), left atrial (LA), and right ventricular (RV) form and function, including parameters derived through two-dimensional, speckle-tracking strain imaging. Another objective was determining if improvements in patients’ echocardiographic indices correlated with patients’ improvement in exercise capacity, as assessed mainly through VO_2_ max.

## 2. Materials and Methods

The design of the RESAFE-HF study has been presented before [36]. RESAFE-HF was an investigator-initiated study, designed and conducted independently as a non-commercial clinical study. A departmental research grant from Vifor Pharma Management Ltd. (Glattbrugg, Switzerland). covered the publication costs, lab materials, and medical equipment conducive to the study. Beginning in June 2020, symptomatic patients with HFrEF, ID, and implanted cardiac implantable electronic devices (CIEDs) about to undergo iron supplementation with intravenous ferric carboxymaltose were eligible for inclusion. The participants in the RESAFE-HF study underwent physical examination, blood testing, echocardiography, 6 min walk testing, cardiopulmonary exercise testing (CPET), quality of life quantification with the Kansas City Cardiomyopathy Questionnaire (KCCQ), and EQ-5D-5L assessment at baseline and after 12 months of follow-up and iron supplementation therapy. The RESAFE-HF study design was approved by the appropriate IRBs and performed in accordance with the set of ethical principles outlined in the Declaration of Helsinki [37].

### 2.1. Inclusion/Exclusion Criteria

All the patients that participated in the RESAFE-HF study were eligible for inclusion in this sub-study. The exclusion criteria for this sub-study were a lack of consent for use of the data beyond the original study or a lack of echocardiographic views as to allow for the quantification of the present study’s echocardiographic endpoints (see below).

### 2.2. Study Workflow

As part of the original study, patients underwent full transthoracic echocardiography on Vivid S6 and Vivid E9 ultrasound machines (GE Healthcare, Chicago, IL, USA) at baseline and at 12 months.

Parasternal long axis views and apical 4-chamber, 5-chamber, 3-chamber, and 2-chamber views lasting at least 3 full cardiac cycles; in addition to spectral Doppler recordings of mitral flow, pulmonary flow, and left ventricular outflow tract flow; as well as tissue doppler imaging (TDI) of the 4-chamber apical view recordings were saved and archived in the EchoPAC software suite v110.1.3 (GE Healthcare, Chicago, IL, USA).

For the purposes of this sub-study, these echocardiographic studies were used to quantify additional structural and functional indices of the LV, LA, and RV (enumerated in Section 2.3). Informed consent for the use of this data was retrieved from all the participants of the RESAFE-HF study. Similar to the original study protocol, all images were analyzed independently by two attending physicians blinded to patient data and study timepoints (baseline vs. 12 months). All quantitative measurements were performed according to the existing recommendations of the American Society of Echocardiography [38]. The mean of the two observations was accepted. In cases where observations differed by more than 10%, a third expert was called upon. Their measurement was considered final.

CPET was performed with breath-by-breath measurements of respiratory gases using the Ultima CPX system (MGC Diagnostics, Saint Paul, MN, USA). The protocol was bicycle ergometry with steadily increasing power (ramping) tailored for each patient according to the algorithm previously validated by Pretto et al. [39]. The test was considered valid only if the peak achieved respiratory exchange ratio (RER) was equal to or higher than 1. The peak VO_2_ achieved was recorded.

### 2.3. Study Endpoints

The endpoints of this sub-study revolved around LV, LA, and RV form and function. LV systolic function was assessed using the LVEF, the LV GLS, and the LVOT VTI, while LV diastolic function was assessed with the E/A ratio (in patients with sinus rhythm), E-wave deceleration time, E/e’ ratio, and diastolic dysfunction grade, as well as the peak early diastolic strain rate. The LV diastolic dysfunction grade was determined based on the flowchart recommended by the 2016 Update from the American Society of Echocardiography and the European Association for patients with reduced LVEF [40]. The grade could not be determined for patients that were in AF during either the baseline or 12-month echocardiographic examination. LV structural information was derived from the LV end-diastolic volume index (LVEDVi) and the LV mass index (LVMi). LV mass was calculated using the Devereux-modified ASE-cube formula: LV mass (g) = 0.8 × {1.04 × [(IVSd + LVIDd + PWTd)^3^ − (LVIDd)^3^]} + 0.6, where IVSd, LVIDd, and PWTd represent the septal thickness, LV internal diameter, and posterior wall thickness in diastole, respectively. LA structure and function were assessed using LAD, LAVi, LAEF, LAFI, and LAS, while RV functional indices that served as endpoints included TAPSE, RV FAC, and RV free wall strain.

### 2.4. Statistical Analysis

Normally distributed continuous variables are reported as mean ± standard deviation and non-normally distributed continuous variables are reported as the median (interquartile range), or the median [interquartile range] when in parentheses. Categorical parameters are reported as the absolute frequency (percentage). If the median of such parameters is 0 (e.g., as with VF episodes per patient per year), they are represented as 5%- or 10%-trimmed mean ± trimmed SD (a choice made with the aim of maximizing readability). Normality was investigated with the Shapiro–Wilk test.

The significance of differences in ordinal and scalar variables was tested with the Wilcoxon signed-rank test, and the McNemar’s test was used for testing differences in paired nominal variables. Missing values due to patient death were imputed with the worst observation of the entire cohort for functional parameters, such as LVEF and strain parameters, while the baseline observation was carried forward for anatomical parameters, such as LAD or LVMi. The significance threshold was set at *p* < 0.05, and the Hochberg–Benjamini procedure was used to control the false discovery rate (FDR) at 20%. The procedure was applied in clusters grouping together variables quantifying LV systolic, LV diastolic, LA, and RV function. For this reason, some *p*-values below 0.05 may not be significant, and this is clearly specified in text and tables. RAA was considered a measure of RV functionality when conducting the Hochberg–Benjamini procedure.

To investigate the relative contribution of the improvement of different cardiac function indices to the amelioration of patients’ exercise tolerance, a backwards, stepwise, multivariate linear regression model was created for each category of indices (LV systolic function, LV diastolic function, LA function, and RA function). The dependent variable was the change in VO_2_ (ΔVO_2_) during follow-up, while independent variables that were evaluated for inclusion were the differences in echocardiographic indices between baseline and after 12 months e.g., ΔLVEF, ΔLAVi, etc. Only variables with a significant (*p <* 0.1) correlation with the ΔVO_2_ were considered candidates for inclusion. The parameters that contributed significantly to each category-specific multivariate model were then inserted into a larger multivariate linear regression model, and stepwise elimination was applied until only significant parameters remained. In the end, the Pratt index was calculated for each significant parameter. The Pratt index allows for the quantification of each parameter’s relative contribution to the ability of the model to explain the variance in the dependent variable: in this case, peak VO_2_ [41,42].

To explore whether improvements in myocardial deformation were influenced by baseline characteristics or the iron repletion status, we conducted subgroup analyses for each of the three echocardiographic strain parameters: left ventricular global longitudinal strain (LV GLS), left atrial strain (LAS), and right ventricular free wall strain (RV FWS). For each patient, the absolute change from baseline to 12 months (Δ = follow-up–baseline for LAS and RV FWS; baseline–follow-up for LV GLS, given its negative scale) was calculated. Subgroups were defined based on clinically relevant variables: age (equal to and above or below median), sex (male vs. female), ischemic vs. dilated cardiomyopathy, and the presence of functional or absolute iron deficiency at baseline (defined as transferrin saturation [TSAT] < 20% or ferritin <100 µg/L, respectively). For each subgroup comparison, the median strain change and 95% confidence interval were calculated, and between-group differences were assessed using the Mann–Whitney U test. The results were visualized using annotated forest plots.

To evaluate the relationship between iron repletion and myocardial functional improvement, we assessed the correlation between changes in iron indices (ΔTSAT and Δferritin) and changes in strain parameters (ΔLV GLS, ΔLAS, ΔRV FWS) using Spearman’s rank correlation coefficient. Scatter plots with regression lines and 95% confidence intervals were generated to visualize these associations. All the analyses were performed using Python (v3.11) with the pandas, seaborn, matplotlib, and scipy libraries.

## 3. Results

**Figure 1:**
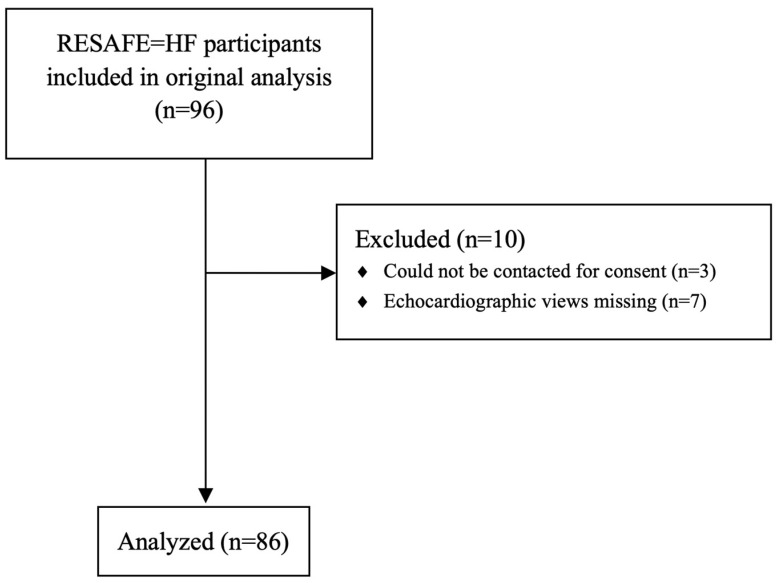
Flow diagram for study participants.

A total of 86 patients out of the 96 patients that participated in the analysis of RESAFE-HF were included in this sub-study (three patients could not be contacted for consent, and seven were missing critical echocardiographic views, Figure 1). The median age was 71.8 (10.7) years, while 71 (83%) patients were male. Approximately half of the patients (44 patients—51%) had ischemic cardiomyopathy, whereas the other half (42 patients—49%) had a history of dilated cardiomyopathy. At baseline, all the patients were on the maximal tolerable treatment, with 85 (99%) receiving beta-blockers, 68 (79%) aldosterone antagonists, and 43 (50%) sacubitril/valsartan. All the patients had implanted CIEDs, with implantable cardioverter-defibrillators (ICDs) being the most common device (44 patients—51%). The patients participating in the study had several comorbidities, with chronic kidney disease being the most common (84% of the patients), followed by a history of any type of atrial fibrillation (70% of the patients) and dyslipidemia (69% of the patients). Table 1 contains a detailed outline of the patients’ baseline characteristics.

Over the course of a 12-month follow-up period, nine (11%) patient deaths directly attributable to HF were recorded, and a further seven (8%) patients expired due to non-HF-related reasons. The patients received an average of 1.35 ± 0.58 g of iron carboxymaltose over the follow-up period, which led to a significant amelioration of patients’ iron status according to all the available metrics (Table 2); at 12 months, 50 patients (58%) no longer fulfilled the ESC criteria for ID (*p* < 0.001). Regarding the endpoints of the original study, most improvements in exercise capacity (i.e., 6 min walking distance and peak VO_2_), quality of life (i.e., total KCCQ score and EQ-5D-5L visual analogue scale), and NT-proBNP hold true in this sub-sample (Supplementary Table S1). All in all, 38 patients had CRT-enabled biventricular pacing, with a satisfactory baseline biventicular pacing rate (median 97% [6.0]), which remained high at 12 months (98.5% [6.8], *p* = 0.266).

### 3.1. LV Structural and Functional Parameters

At baseline, the mean LVEF was 29.3 ± 7.8%, and the median LV GLS was −7.89% (3.8). After 12 months of iron supplementation therapy, the mean LVEF increased to 32.5 ± 10.6 (*p* < 0.001), while the LV GLS improved to −8.62% (4.7) (*p* = 0.001). Additionally, the median LVEDVi decreased significantly during follow-up, from 89.9 mL/m^2^ (39.5) to 87.8 mL/m^2^ (52.3) (*p* = 0.027).

**Figure 2:**
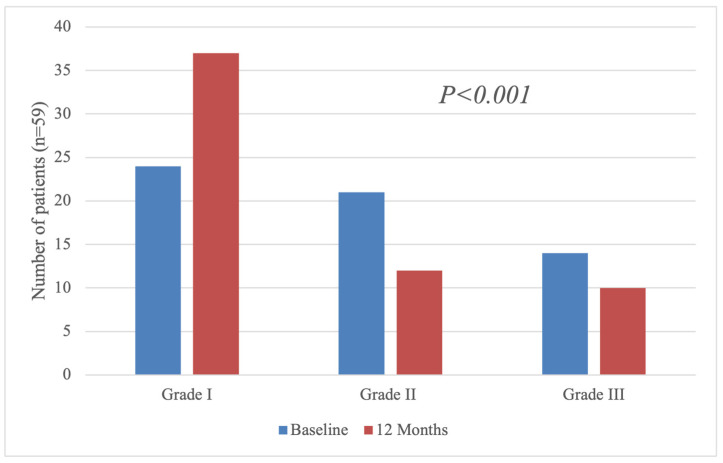
Diastolic dysfunction grades of patients at baseline and after 12 months of iron supplementation.

Regarding LV diastolic function, the diastolic dysfunction grade could be assessed in 59 (69%) patients who were in sinus rhythm or DDD(R) pacing mode for both the baseline and 12-month echocardiograms. These data indicate a significant improvement in patients’ diastolic function—over the course of follow-up, 16 patients (27% of patients in sinus rhythm) with detectable diastolic dysfunction reverted to Grade I (*p* < 0.017, Figure 2). This improvement is also observable in the constituents of this classification system; patients’ median E/e’ ratio decreased to 13 (8.5) at 12 months compared to 15.1 (7.3) (*p* < 0.017), while the E-wave deceleration time increased to 188 ms (86) from 173 ms (65) at baseline (*p* < 0.001). The full range of anatomic and functional indices for the LV can be found in Table 3. The improvements in ferritin and TSAT did not, per se, correlate with improvements in the median LV GLS (Supplementary Figure S4). A subgroup analysis of the change in LV GLS from baseline to 12 months demonstrated consistent improvement across patient categories, including age, sex, etiology of cardiomyopathy, and presence of absolute or functional iron deficiency at baseline with no statistically significant interactions (Supplementary Figure S1).

### 3.2. LA Structural and Functional Parameters

The median patient LAVi decreased significantly from 37.4 mL/m^2^ (25.1) to 32 mL/m^2^ (19.7) after 12 months of follow-up (*p* < 0.001). An increase in the median patient LAFi was also noted after iron supplementation (20.4 [30.3] at baseline compared to 21.7 [37] at 12 months, *p* = 0.009). Similarly, the peak LA strain (reservoir strain) improved significantly during follow-up, with the median LA strain reaching 15.1% (11.9) at 12 months compared to 13.9% (12.5) at baseline (*p* = 0.02). All of the LA indices are presented in Table 4. The improvements in ferritin and TSAT did not, per se, correlate with improvements in the median LA strain (Supplementary Figure S4). A subgroup analysis of the change in LA strain from baseline to 12 months demonstrated consistent improvement across patient categories, including age, sex, etiology of cardiomyopathy, and presence of absolute or functional iron deficiency at baseline with no statistically significant interactions (Supplementary Figure S2).

### 3.3. RV Structural and Functional Indices

A significant reduction in RV size was observed, with the median RV EDA decreasing to 17 cm^2^ (6.5) from 18.6 cm^2^ (8.2) at baseline (*p* < 0.001). Significant improvement was noted in most of the functional RV indices, with the mean patient TAPSE increasing to 17.6 mm ± 3.5 from 16.4 cm ± 3.6 (*p* = 0.008) and the median TDI S’ wave velocity reaching 9.2 ± 2.1 cm/sec at 12 months compared to a baseline of 8.2 ± 1.9 (*p* < 0.001). RV free wall strain also improved significantly; the median value at baseline was 16.9 ± 4.5% and 19.4 ± 5.4% at 12 months (*p* < 0.001). All the RV indices are presented in detail in Table 5. Right atrial area (RAA) was the only right atrial parameter to be examined in this sub-study, and here too a significant improvement was observed, as the median RAA dropped to 15 cm^2^ (8) from 16.2 cm^2^ (7.3) at baseline (*p* = 0.028). The improvements in ferritin and TSAT did not, per se, correlate with improvements in the median RV free wall strain (Supplementary Figure S4). A subgroup analysis of the change in RV free wall strain from baseline to 12 months demonstrated consistent improvement across the patient categories, including age, sex, etiology of cardiomyopathy, and presence of absolute or functional iron deficiency at baseline with no statistically significant interactions (Supplementary Figure S3).

### 3.4. Improvement in LV and RV Function as a Mediator of Exercise Capacity Improvement

**Figure 3:**
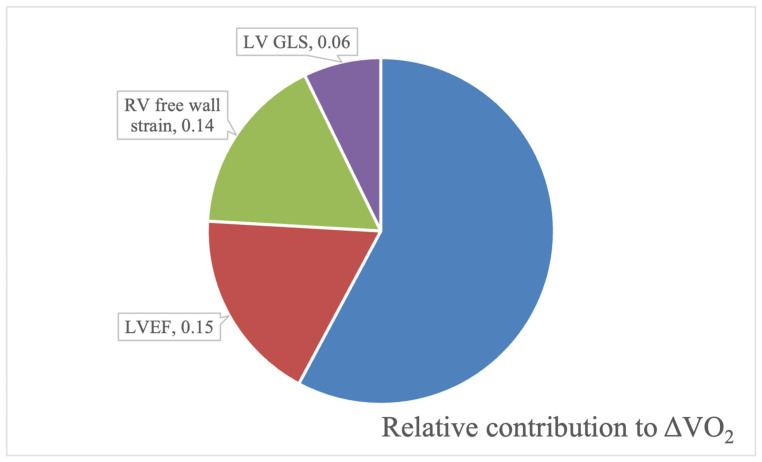
The relative contribution of left ventricular ejection fraction (LVEF), left ventricular global longitudinal strain (LV GLS), and right ventricular free wall strain to the improvement of peak VO_2_. The proportion of unaccounted variance was based on R^2^ values; the relative contribution of explained variance was based on each parameter’s Pratt index.

The patients participating in RESAFE-HF experienced a significant improvement in peak VO_2_ after 12 months of intravenous iron supplementation, and this holds true in this subgroup as well (excluding nine patients who could not complete the baseline and/or 12-month CPET examination due to musculoskeletal problems or dyspnea at rest, the peak VO_2_ was 11.3 ± 3.2 mL/min/kg compared to 12.1 ± 4.1 mL/min/kg at 12 months, *p* < 0.001). As described in the Methods Section, a multivariate model was developed for each category of the echocardiographic indices to examine their contribution to VO_2_ improvement (abbreviated to ΔVO_2_) (Supplementary Tables S2–S5). The best-performing indices of each category were candidates for inclusion in a general multivariate linear regression model. The model’s characteristics can be found in Table 6, and the relative contribution of each parameter (its Pratt index) is graphically presented in Figure 3. Overall, improvements in LVEF (*p* < 0.001), RV FWS (*p* < 0.001), and LV GLS (*p* = 0.01) were all independent predictors of an increase in peak VO_2_, with ΔLVEF and ΔRV GLS being by far the strongest parameters in this regard.

## 4. Discussion

The aim of this sub-study of RESAFE-HF was to investigate the effect of intravenous iron supplementation with FCM on anatomical and functional indices of the LV, LA, and RV. The main findings are as follows:

- LV form and systolic function improved significantly after iron supplementation, evidenced by a significant reduction in LVEDVi and improvements in LVEF and LV GLS. Additionally, LV end-diastolic pressures significantly improved, as reflected by the overall LV diastolic dysfunction grade, as well as E/e and E/A, which were evaluated separately.
- LA size and mechanics improved, as reflected in changes in LAVi, LAFI, and peak LA strain (reservoir strain).
- RV functional indices improved—a significant reduction in RV EDA and an increase in the median RV free wall strain and RV TDI S’ wave velocity was observed.
- The improvements in LV and RV systolic function (more specifically LVEF, LV GLS, and RV free wall strain) were independent predictors of an increase in the peak VO_2_.

To our knowledge, it is the first time such a comprehensive panel of echocardiographic indices covering all aspects of cardiac function have been evaluated simultaneously in patients with HFrEF and ID undergoing iron supplementation with IV FCM. Our results indicate that all aspects of cardiac function exhibited improvement, while FCM’s beneficial effects on the patients’ exercise capacity may be mediated through improvements in RV and LV systolic function.

RCTs, such as FAIR-HF [32], FERRIC-HF [43], CONFIRM-HF [31], and EFFECT-HF [29], offer a multitude of evidence to substantiate the positive influence of IV FCM supplementation on HFrEF patients’ hospitalization rate, exercise capacity, quality of life, and prognostic biomarkers, such as NT-proBNP [28,44]. However, the effect of iron therapy on echocardiographic indices in HFrEF and ID has not been the focus of attention, as none of the aforementioned trials included an echocardiographic endpoint.

What data exist align with our findings, suggesting LV and RV function improvements secondary to intravenous iron supplementation. Martens et al. investigated the effect of IV FCM on the LVEDV and LVEF of patients with HFrEF and ID in the IRON-CRT trial, reporting a significant increase in LVEF to the tune of 4.22% on average [45]. In a later study, Martens et al. investigated the effect of IV FCM on RV functionality in the same group of patients. TAPSE and RV FAC exhibited significant improvements that correlated with the increase in LVEF [46]. Our findings align with both studies, as patients participating in this sub-study of RESAFE-HF experienced a significant improvement in LVEF, TAPSE, and several other LV and RV indices.

A number of cardiac magnetic resonance (CMR) studies investigated the effect of IV FCM on HFrEF and ID patients’ LV functional indices. Nunez et al. [47] were actually able to observe the restitution of myocardial iron stores as a decrease in T2* sequence, which was associated with a non-significant trend towards LVEF improvement—the difference in the median LVEF after approximately 1.5 months was +4.8%, but the small study size (eight patients) limited the impact of these findings. An expanded trial with 53 HFrEF patients with ID undergoing iron therapy with IV FCM corroborated the original observation that the myocardial T2* sequence decreases in response to FCM as a sign of myocardial iron restitution [47]. However, the median +4% increase in LVEF among the patients in the FCM arm compared to the control arm once again failed to reach statistical significance. The RESAFE-HF patients, on average, experienced an improvement in LVEF of similar magnitude. However, our study was adequately powered to achieve statistical significance.

The findings of this study also shed light on the mechanism through which the restitution of iron stores improves exercise capacity in this group of patients. Combined, the improvements in LVEF, LV GLS, and RV free wall strain accounted for 42% of the variation in peak VO_2_ change between baseline and 12 months. This further establishes that ID is not an ancillary concern but a core pathophysiologic pillar of HFrEF with deleterious effects on LV and RV function. Intravenous iron supplementation with FCM leads to improvement of right and left cardiac function and, ultimately, to a higher peak VO_2_, reflecting the increased exercise capacity. Our findings are thus compatible with recent evidence indicating that LV GLS correlates with exercise capacity in HFrEF patients [48].

Several of the parameters that improved in our study, including the diastolic dysfunction grade, left atrial volume index (LAVi), left atrial strain, TAPSE, and RV free wall strain, have been previously shown to independently predict mortality and adverse outcomes in patients with HFrEF, underscoring the potential clinical relevance of the observed echocardiographic changes [49].

Naturally, this sub-study has several limitations. The lack of a control arm makes it difficult to appraise the effect of confounding factors that may have influenced the patients’ echocardiographic indices in addition to iron supplementation. However, the patients were already on the maximal tolerated treatment and had not recently had an acute-on-chronic HF episode, as per the exclusion criteria, minimizing the potential effect of statistical epiphenomena, such as regression to the mean, on the study results [50]. As all the patients in this sub-study were required to have an implanted cardiac device (ICD, CRT, or pacemaker) per the original RESAFE-HF protocol, the findings may not be fully generalizable to HFrEF patients without device therapy, although such devices represent a key component of guideline-directed medical therapy in eligible individuals. Additionally, this study relies on transthoracic echocardiography (TTE) to quantify both strain and conventional functional parameters. While TTE remains a widely accepted and accessible tool for cardiac assessment, its accuracy can be influenced by image quality, operator expertise, and inter-vendor variability. Strain imaging in particular is sensitive to tracking quality and vendor-specific post-processing algorithms, while traditional echocardiographic parameters are also subject to interpretive variability and loading conditions [51,52]. Additionally, although several of the observed changes reached statistical significance, their absolute magnitudes were relatively small. These factors should be considered when interpreting the echocardiographic findings and their potential clinical relevance. SGLT2i penetration among the sample was low at baseline and remained low throughout the study as most study visits were concluded prior to their incorporation into the ESC guidelines for chronic heart failure treatment [25]. As a result, potential interactions could not be investigated as part of this study [53]. Tracking patients’ iron content through CMR over the follow-up period would also have been useful as an additional indication that IV FCM supplementation directly acts on LV and RV functionality. Alas, this was not feasible at the time.

## 5. Conclusions

In conclusion, this sub-study of the RESAFE-HF registry study examined various echocardiographic indices of LV, LA, and RV form and function in patients with HFrEF and ID undergoing intravenous iron therapy with FCM. Both conventional and strain-derived parameters signaled a significant amelioration in patients’ cardiac function. In the form of LVEF, LV GLS, and RV free wall strain gains, this partial restoration of LV and RV function was independently and significantly associated with the concomitant improvement in exercise capacity, as quantified through peak VO_2_, further highlighting the unique importance of intravenous iron therapy in this group of patients. Further studies will be necessary to establish the causality of this association, preferably including simultaneous non-invasive quantification of myocardial iron content.

## Figures and Tables

**Table 1:** Baseline characteristics of study participants.

Variable	All Patients (n = 86)
Age (y)	71.8 (10.7)
Sex (male, %)	71 (83%)
BMI (kg/m^2^)	29.1 (5.4)
NYHA Class	
I	0 (0%)
II	41 (48%)
III	40 (46%)
IV	5 (5%)
Ischemic (%)	44 (51%)
Pacemaker	4 (5%)
ICD	44 (51%)
CRT-P	6 (7%)
CRT-D	32 (37%)
Diabetes	37 (43%)
Stroke	13 (15%)
CKD	72 (84%)
Dyslipidemia	59 (69%)
AF	60 (70%)
Paroxysmal AF	35 (58%)
Permanent AF	25 (42%)
ACEi or ARB or ARNi	63 (73%)
ACE or ARB only	20 (23%)
ARNi	43 (50%)
B-blockers	85 (99%)
Aldosterone antagonists	68 (79%)
SGLT2i	6 (7%)
Amiodarone	41 (48%)

BMI—body mass index; NYHA—New York Heart Association; ICD—implantable cardioverter defibrillator; CRT-P—cardiac resynchronization therapy (pacing); CRT-D cardiac resynchronization therapy device with cardioverter-defibrillator capability; CKD—chronic kidney disease; AF—atrial fibrillation; ACEi—angiotensin-converting enzyme inhibitors; ARB—angiotensin receptor blockers; ARNi—angiotensin receptor–neprilysin inhibitor; SGLT2i—Sodium/glucose cotransporter-2 inhibitors.

**Table 2:** Iron-related outcomes at baseline and 12 months.

Variable	Baseline	12 Months	*p*-Value
Ferritin (μg/L)	56.4 (63.7)	147.1 (124.6)	<0.001
TSAT (%)	19 (10.8)	24.4 (12.3)	<0.001
Functional Iron Deficiency (%)	53 (62%)	28 (33%)	<0.001
Absolute Iron Deficiency (%)	74 (86%)	26 (30%)	<0.001
ESC-defined Iron Deficiency (%)	86 (100%)	36 (42%)	<0.001

**Table 3:** Outcomes related to LV form and function at baseline and 12 months (n = 86 except where stated).

Variable	Baseline	12 Months	*p*-Value
LV anatomical indices			
LVEDVi (mL/m^2^)	89.9 (39.5)	87.8 (52.3)	0.027
LVMi (kg/m^2^)	119.3 (40.3)	120.7 (39.7)	0.82
LV systolic function indices			
LVEF (%)	29.3 ± 7.8	32.5 ± 10.6	<0.001
LVOT VTI (m)	15.6 (5.1)	17.4 (6.6)	<0.001
LV GLS (%)	−7.89 (3.8)	−8.62 (4.7)	0.001
LV diastolic function indices			
Diastolic dysfunction grade (I/II/III) (n = 59)	24 (41%)/21 (36%)/14 (23%)	37 (63%)/12 (20%)/10 (17%)	0.017
E’ wave velocity (cm/s)	0.78 (0.45)	0.73 (0.52)	0.023
E-wave deceleration time (msec)	173 (65)	188 (86)	0.001
E/A ratio (n = 59)	15.1 (7.3)	13 (8.5)	0.017
LV peak early diastolic strain rate (s^−1^)	0.43 (0.21)	0.48 (0.22)	0.021

LVEDVi—left ventricular end-diastolic volume index; LVMi—left ventricular mass index; LVEF—left ventricular ejection fraction; LVOT VTI—left ventricular outflow tract velocity time integral; LV GLS—left ventricular global longitudinal strain.

**Table 4:** Outcomes related to LA form and function at baseline and 12 months.

Variable	Baseline	12 Months	*p*-Value
LAD (mm)	44.4 ± 7.6	43.6 ± 7.5	0.117
LAVi (mL/m^2^)	37.4 (25.1)	32 (19.7)	<0.001
LAEF (%)	50 (39.8)	44.8 (34)	0.096
LAFI	20.4 (30.3)	21.7 (37)	0.009
LA Strain	13.9 (12.5)	15.1 (11.9)	0.02

LAD—left atrial diameter; LAVi—left atrial volume index; LAEF—left atrial emptying fraction; LAFI—left atrial function index; LA—left atrial; strain—peak systolic left atrial strain (reservoir).

**Table 5:** Outcomes related to right heart form and function at baseline and 12 months.

Variable	Baseline	12 Months	*p*-Value
RV EDA (mm^2^)	18.6 (8.2)	17 (6.5)	<0.001
RAA (mm^2^)	16.2 (7.3)	15 (8)	0.028
TAPSE (mm)	1.64 ± 0.36	1.76 ± 0.35	0.008
RV FAC (%)	40.1 ± 11.8	42.5 ± 12.7	0.111
RV TDI S’ velocity (cm/s)	8.2 ± 1.9	9.2 ± 2.1	<0.001
RV free wall strain (%)	16.9 ± 4.5	19.4 ± 5.4	<0.001

RV—right ventricular; EDA—end-diastolic area; RAA—right atrial area; TAPSE—tricuspid annular plane systolic excursion; FAC—fractional area change; TDI—tissue doppler imaging.

**Table 6:** Characteristics of a multivariate linear regression model developed to investigate associations between ΔVO_2_ and improvements in echocardiographic indices.

Model Parameter	Beta Coefficient	Standard Error	t	*p*-Value	95% Confidence Intervals	Pratt Index
LVEF	0.15	0.03	4.89	<0.001	0.09–0.21	0.15
RV free wall strain	0.19	0.05	3.74	<0.001	0.09–0.3	0.14
LV GLS	0.37	0.14	2.63	0.01	0.09–0.65	0.06
R^2^ value	Variables excluded through stepwise elimination:					
0.42	LVOT VTI, E’ wave deceleration time, LAEF, LAFI					

LVEF—left ventricular ejection fraction; RV—right ventricular; LV—left ventricular; GLS—global longitudinal strain.

## Data Availability

Data is available upon request.

## Supplementary Materials

The following supporting information can be downloaded at https://www.mdpi.com/article/10.3390/diagnostics15151941/s1. Table S1: RESAFE-HF endpoints in the group of patients participating in this sub-study. Table S2: Characteristics of a multivariate linear regression model developed to investigate associations between ΔVO_2_ and improvements in echocardiographic indices of LV systolic function. Table S3: Characteristics of a multivariate linear regression model developed to investigate associations between ΔVO_2_ and improvements in echocardiographic indices of LV diastolic function. Table S4: Characteristics of a multivariate linear regression model developed to investigate associations between ΔVO_2_ and improvements in LA echocardiographic indices. Table S5: Characteristics of a multivariate linear regression model developed to investigate associations between ΔVO_2_ and improvements in echocardiographic indices of RV function. Figure S1: Change in Left Ventricular Global Longitudinal Strain (LV GLS) over 12 Months by Subgroup. Figure S2: Change in Right Ventricular Free Wall Strain (RV FWS) over 12 Months by Subgroup. Figure S4: Correlation Between Changes in Iron Parameters and Myocardial Strain Over 12 Months.

## Abbreviations

The following abbreviations are used in this manuscript:

AF	atrial fibrillation
CPET	cardiopulmonary exercise testing
CRT	cardiac resynchronization therapy
CMR	cardiac magnetic resonance
CIED	cardiac implantable electronic device
E/A	ratio of early (E) to late (A) diastolic mitral inflow velocities
E/e′	ratio of early mitral inflow velocity to early mitral annular velocity
ESC	European Society of Cardiology
FCM	ferric carboxymaltose
FDR	false discovery rate
GLS	global longitudinal strain
HF	heart failure
HFrEF	heart failure with reduced ejection fraction
ID	iron deficiency
ICD	implantable cardioverter-defibrillator
IRB	institutional review board
LA	left atrium/atrial
LAD	left atrial diameter
LAEF	left atrial emptying fraction
LAFI	left atrial function index
LAS	left atrial strain
LAVi	left atrial volume index
LV	left ventricle/ventricular
LVEDVi	left ventricular end-diastolic volume index
LVEF	left ventricular ejection fraction
LVMi	left ventricular mass index
LVOT VTI	left ventricular outflow tract velocity time integral
NT-proBNP	N-terminal pro-B-type natriuretic peptide
RA	right atrium/atrial
RAA	right atrial area
RER	respiratory exchange ratio
RV	right ventricle/ventricular
RV FAC	right ventricular fractional area change
RV FWS	right ventricular free wall strain
SD	standard deviation
TAPSE	tricuspid annular plane systolic excursion
TDI	tissue doppler imaging
TSAT	transferrin saturation
TTE	transthoracic echocardiography
VO_2_	oxygen consumption (often peak VO_2_ = peak oxygen uptake)
