# Supplementary material for: Changes in Cardiac Function and Exercise Capacity Following Ferric Carboxymaltose Administration in HFrEF Patients with Iron Deficiency
Source: Diagnostics (Basel). 2025 Aug 2;15(15):1941. doi: 10.3390/diagnostics15151941 (PMC12345828; doi:10.3390/diagnostics15151941)
Supplement: Supplementary file 1 [file diagnostics-15-01941-s001.zip › diagnostics-3744163-supplementary.pdf]

**Supplementary Table S1.** RESAFE-HF endpoints in the group of patients participating in this sub-study (n=86 except when stated).

| Variable | Units/ Notes | Baseline | 12 months | *P*-value |
|---|---|---|---|---|
| Primary Outcome | | | | |
| Composite Iron Outcome | Ferritin>50µg/L, TSAT>20% & Hgb>12g/dL | 21 (24%) | 51 (59%) | **<0.001** |
| Secondary outcomes | | | | |
| Iron outcomes | | | | |
| ESC Iron Deficiency criteria | Ferritin>300µg/L or Ferritin >100µg/L & TSAT >20% | 0 (0%) | 50 (58%) | **<0.001** |
| Hemoglobin | g/dL | 13±1.47 | 13.1±1.85 | 0.167 |
| TSAT | % | 19 (10.8) | 24.4 (12.3) | **<0.001** |
| Ferritin | ng/mL | 56.4 (63.7) | 147.1 (124.6) | **<0.001** |
| Arrhythmic outcomes | | | | |
| VT/VF episodes | per 100 patient years | 52±159 | 22±71 | 0.088 |
| nsVT episodes | per 100 patient years | 355±738 | 106±221 | **0.001** |
| Appropriate shocks (n=76) | Per 100 patient years ICD-pts only | 12±50 | 3±18 | 0.327 |
| Appropriate therapies (n=76) | Per 100 patient years ICD-pts only | 38±148 | 11±46 | 0.118 |

| Variable | Units/ Notes | Baseline | 12 months | *P*-value |
|---|---|---|---|---|
| Exercise capacity outcomes | | | | |
| 6MWD (n=79) | m | 431 (113) | 469 (134) | **<0.001** |
| Peak $VO_2$ | mL/kg/min | 11.3±3.2 | 12.1±4.1 | **<0.001** |
| Biomarker | | | | |
| NT-proBNP | ng/mL | 723 (2354) | 309 (1596) | **<0.001** |
| Quality of life outcomes | | | | |
| EQ-5D-5L VAS | % | 60 (20) | 68 (22) | **<0.001** |
| KCCQ total | % | 69.9 (25.7) | 81.3 (24.8) | **<0.001** |

**Supplementary Table S2.** Characteristics of a multivariate linear regression model developed to investigate associations between $\Delta VO_2$ and improvements in echocardiographic indices of LV systolic function.

| Model parameter | Beta coefficient | Standard Error | t | *P*-value | 95% confidence intervals | Pratt index |
|---|---|---|---|---|---|---|
| LVEF | 0.13 | 0.03 | 4.15 | <0.001 | 0.07-0.2 | 0.15 |
| LV GLS | 0.35 | 0.14 | 2.46 | 0.016 | 0.07-0.64 | 0.06 |
| LVOT VTI | 0.17 | 0.06 | 2.96 | 0.004 | 0.06-0.29 | 0.15 |
| $R^2$ value | Variables excluded through stepwise elimination: | | | | | |
| 0.38 | LVEDVi | | | | | |

**Supplementary Table S3.** Characteristics of a multivariate linear regression model developed to investigate associations between $\Delta VO_2$ and improvements in echocardiographic indices of LV diastolic function.

| Model parameter | Beta coefficient | Standard Error | t | *P*-value | 95% confidence intervals | Pratt index |
|---|---|---|---|---|---|---|
| E' wave deceleration time | 0.006 | 0.003 | 2.3 | 0.024 | 0.001-0.012 | 0.02 |
| $R^2$ value | Variables excluded through stepwise elimination: | | | | | |
| 0.065 | E/e', Peak early diastolic strain rate | | | | | |

**Supplementary Table S4.** Characteristics of a multivariate linear regression model developed to investigate associations between $\Delta VO_2$ and improvements in LA echocardiographic indices.

| Model parameter | Beta coefficient | Standard Error | t | *P*-value | 95% confidence intervals | Pratt index |
|---|---|---|---|---|---|---|
| LAEF | -0.03 | 0.01 | -2.7 | 0.008 | -0.06 - -0.009 | 0.02 |
| LAFI | 0.04 | 0.01 | 2.6 | 0.011 | 0.008 – 0.062 | 0.01 |
| $R^2$ value | Variables excluded through stepwise elimination: | | | | | |
| 0.117 | LAVi, LA Strain | | | | | |

**Supplementary Table S5.** Characteristics of a multivariate linear regression model developed to investigate associations between $\Delta VO_2$ and improvements in echocardiographic indices of RV function.

| Model parameter | Beta coefficient | Standard Error | t | *P*-value | 95% confidence intervals | Pratt index |
|---|---|---|---|---|---|---|
| RV free wall strain | 0.25 | 0.05 | 4.72 | <0.001 | 0.15-0.36 | 0.34 |
| $R^2$ value | Variables excluded through stepwise elimination: | | | | | |
| 0.226 | RV EDA, TAPSE, RV TDI S’ velocity | | | | | |

**Supplementary Figure S1.** Change in Left Ventricular Global Longitudinal Strain (LV GLS) over 12 Months by Subgroup

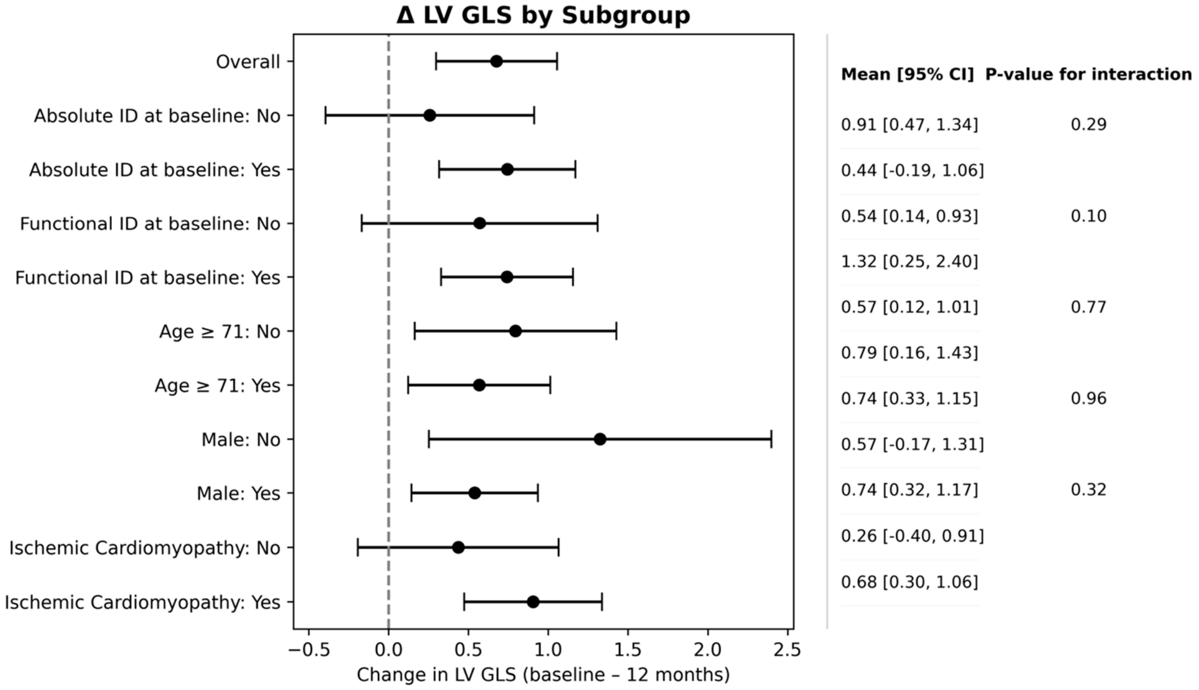


Forest plot showing mean change in LV GLS (baseline – 12 months) with 95% confidence intervals across key clinical subgroups. Negative values reflect improvement in strain.

**Supplementary Figure S2.** Change in Left Atrial Strain (LA Strain) over 12 Months by Subgroup

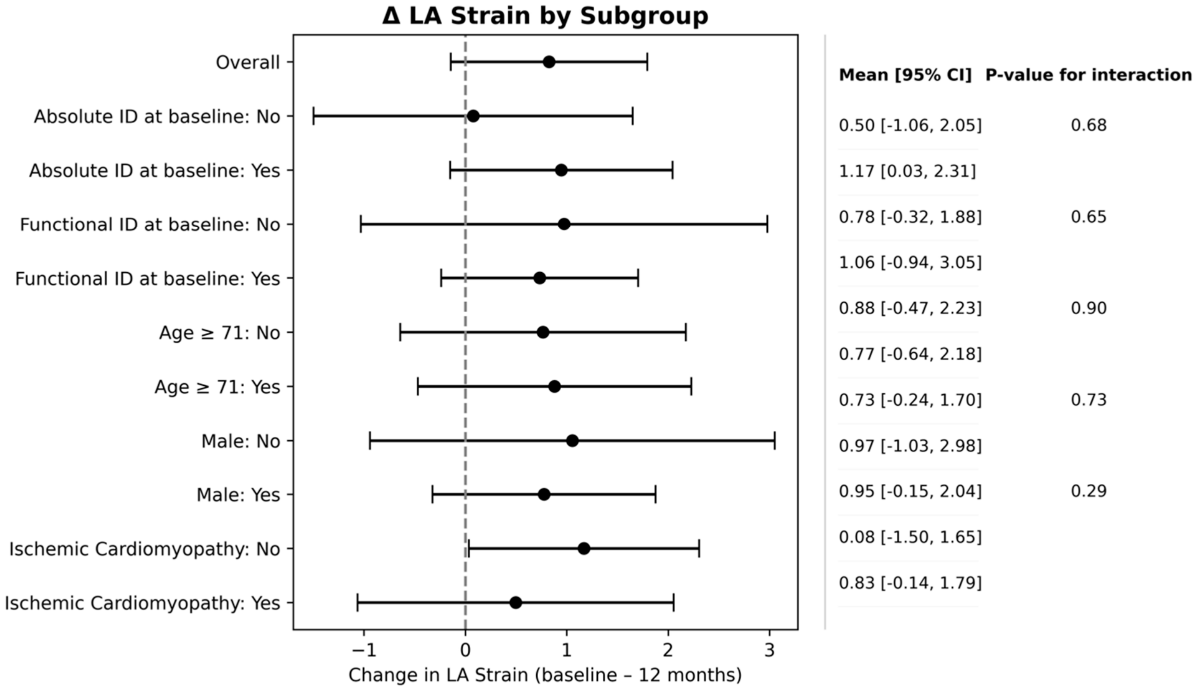


Forest plot illustrating mean change in LA strain with 95% confidence intervals, stratified by baseline clinical characteristics. Higher values indicate improvement. Please note that although the overall change in LA strain was statistically significant ($p < 0.05$, Wilcoxon signed-rank test), the mean estimate is accompanied by a wide 95% confidence interval that includes zero. This reflects asymmetry and variability in individual responses, as well as the use of normal approximation to compute confidence intervals around non-normally distributed paired differences. The statistical test, which accounts for within-subject directionality, remains valid and more sensitive for detecting change than visual overlap with 0.

**Supplementary Figure S3.** Change in Right Ventricular Free Wall Strain (RV FWS) over 12 Months by Subgroup

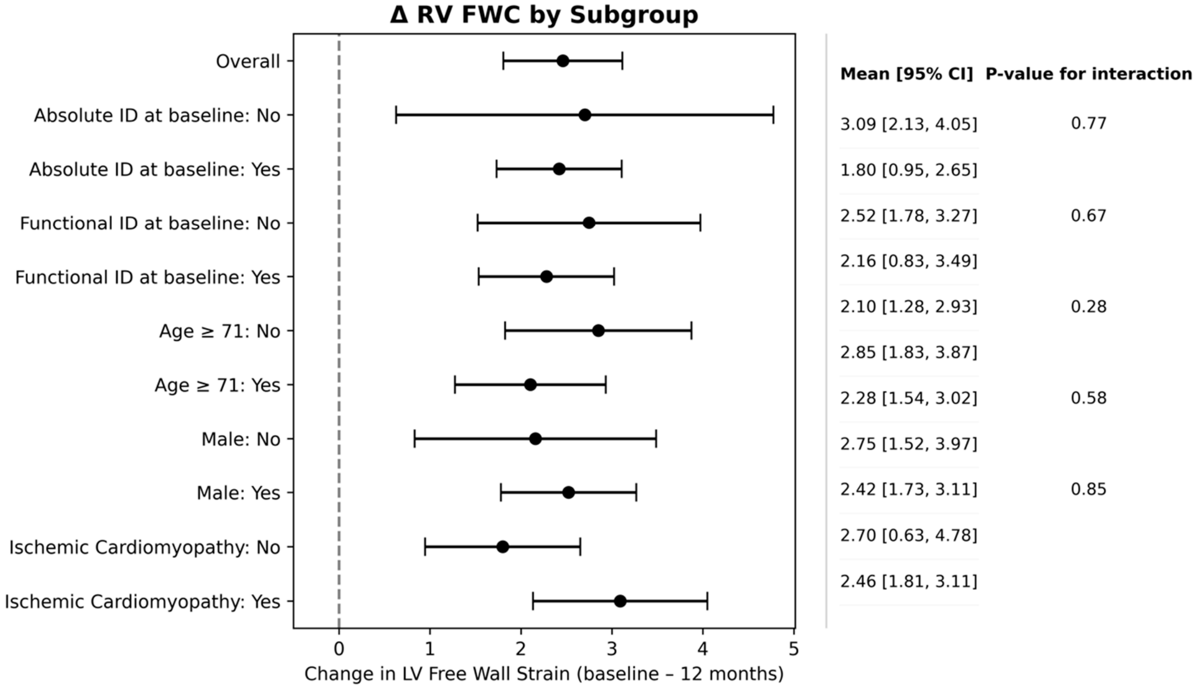


Mean change in RV free wall strain (baseline – 12 months) with 95% confidence intervals, stratified by iron status, age, sex, etiology, and other baseline variables.

**Supplementary Figure S4.** Correlation Between Changes in Iron Parameters and Myocardial Strain Over 12 Months

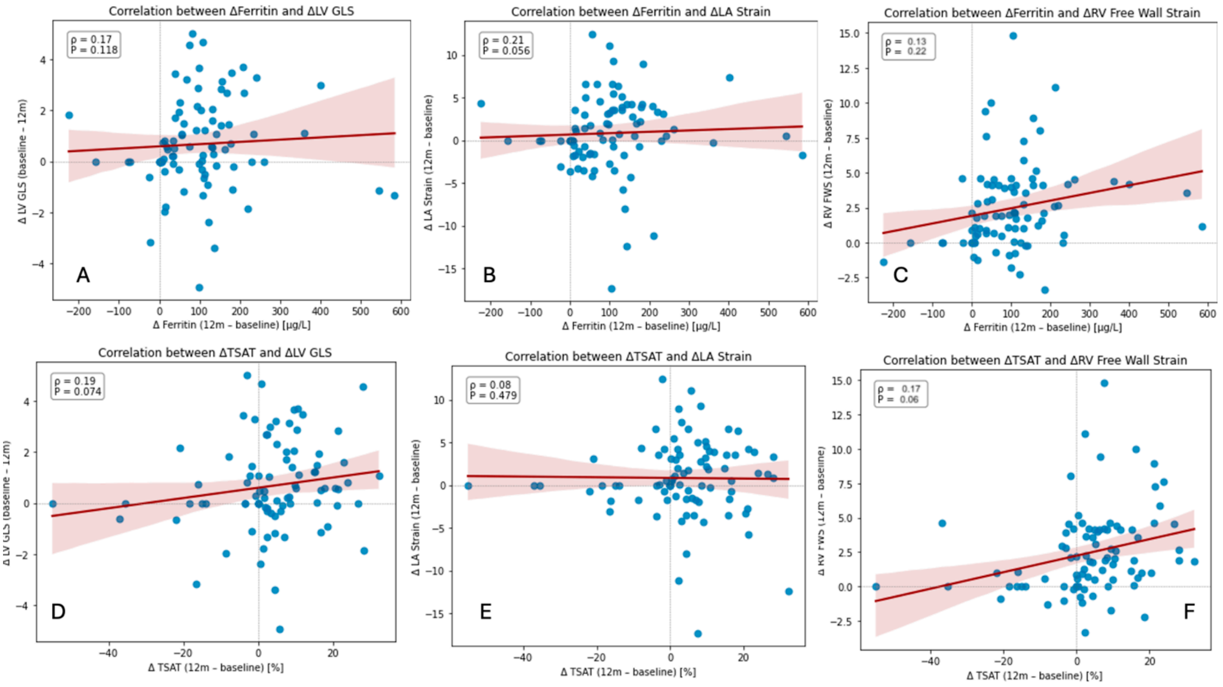


Shown are scatter plots illustrating the relationship between changes in iron status markers—ferritin (top row: panels A–C) and transferrin saturation (TSAT; bottom row: panels D–F)—and corresponding changes in myocardial deformation parameters after 12 months of follow-up.

- Panels A–C: Association of ΔFerritin with changes in left ventricular global longitudinal strain (LV GLS), left atrial strain (LA strain), and right ventricular free wall strain (RV FWS), respectively.
- Panels D–F: Association of ΔTSAT with the same three cardiac strain measures.

Spearman's correlation coefficients (ρ) and corresponding *P* values are displayed in each panel. Linear regression lines with 95% confidence intervals (shaded area) are overlaid. Positive changes in LV GLS, LA strain, and RV FWS reflect improved myocardial deformation.
